# Preparation Method of Porous Dressing Materials Based on Butyric-Acetic Chitin Co-Polyesters

**DOI:** 10.3390/ma11122359

**Published:** 2018-11-23

**Authors:** Zbigniew Draczynski, Beata Kolesinska, Ilona Latanska, Witold Sujka

**Affiliations:** 1 Department of Material and Commodity Sciences and Textile Metrology, Lodz University of Technology, Zeromskiego 116, 90-924 Lodz, Poland; zbigniew.draczynski@p.lodz.pl; 2 Institute of Organic Chemistry, Lodz University of Technology, Zeromskiego 116, 90-924 Lodz, Poland; beata.kolesinska@p.lodz.pl; 3 Tricomed S.A., Lodz, Swietojanska 5/9, 93-493 Lodz, Poland; Ilona.Latanska@tricomed.com

**Keywords:** wound healing, chitin ester, porous materials, salt leaching

## Abstract

A method for obtaining highly porous materials in the form of film, based on the butyric-acetic chitin co-polyesters, containing 90% of butyryl and 10% of acetyl groups, was developed. The highly porous films, with thickness up to 0.11 mm, were obtained by two methods: (a) pouring 5% BAC 90/10 solution in ethanol on the layer of solid salts (porophor agent) which after solidification was eluted with water; (b) application of the suspension of porophor agent in BAC 90/10 solution in the solvent mixture with density similar to bulk porophor agent. In the final stage, the materials were obtained with porosity up to 95–99% and tensile strength 5 cN, which can be used as an active layer of medical dressings. The optimised procedure was used in the production of porous medical dressings (Medisorb) on an industrial scale. In the industrial method, NaCl was used as a porophor agent in the solid form and as a 3% solution in polymer. The final materials were characterised by porosity and other functional parameters at the level recommended for medical dressings. Medisorb series materials do not show in vitro cytotoxic activity.

## 1. Introduction

Modern dressing materials used to obtain wound dressings should not only protect the injured tissue from external factors but also enhance the recovery process and temporarily should have the ability to overtake the activity and function of damaged tissue. Dressing materials should stimulate damaged tissue to faster recovery and regeneration by supplying adhesion and finally multiplying tissue cells in the damaged zone. The materials most valuable in this aspect are resorbable polymers, especially biopolymers. The benefit from use of resorbable materials characterised by specific decomposition time in a living organism stems from the fact that they are biologically active and function only for a precisely established time, after which they become biodegradable to compounds harmless to organisms. From the perspective of the regeneration process and patient’s comfort, the use of resorbable dressing materials does not require their change, but after their total resorption, a new dressing application is recommended, which does not hamper the wound healing process and does not damage the new-built tissue. The best characterised resorbable materials used in the production of dressings are biodegradable and bioresorbable synthetic polymers: poly-lactide and poly-glycol and their copolymers, poly-ε-caprolactone [1,2] and polymers of natural origin, e.g., chitin, chitosan, calcium alginate, and their ester and ether derivatives [3,4,5].

Chitin (poly-[1,4]-*N*-acetyl-D-glucose amine) and their derivatives are commonly known and their bioactivity have been confirmed by many tests [6,7,8]. Chitin is non-toxic and biocompatible in contact with tissue and blood, and does not evoke allergic reactions. It influences the macrophage activity, increases the intensity of tissue hypertrophic granulation and growth of small blood vessels in the wound zone, which fastens the healing process of the wound [8,9,10]. Chitin is an easily accessible biopolymer, and it is estimated that the annual natural reproducibility of chitin by biosynthesis is 2–3 billion tons. The most significant chitin drawback is its almost total insolubility in water, dilute acids and bases, as well as organic solvents. As a result, the practical use of chitin requires its transformation in urethane, ether, or ester derivatives [11,12,13]. Chitin esters, taking into consideration their physio-chemical, mechanical, and biological properties, are materials often used in medicine [14,15,16]. Selection of structure and the properties of acyl derivatives used for chitin esterification is connected with the expected characteristics of modified biopolymer; which means that not all chitin esters find their practical use.

One of the known and used methods of modulation of chitin chemical properties is the esterification leading to carboxymethylchitin or *N*,*N*-dicarboxymethyl chitosan derivatives. The method is based on the esterification of chitin with monochloroacetic or monochloropropanoic acid and subsequent substitution of chlorine with the hydroxyl group in the presence of sodium hydroxide. The modification leads to the loosening of the supramolecular structure of chitin and the obtaining of the chitin derivatives soluble in water [17].

A different approach to chitin modification was based on the preparation of the ester of chitin, which is well soluble in organic solvents, allowing the manufacturing of different materials. One of the chitin derivatives known to dissolve in organic solvents is the dibutyrylchitin ester (DBC) [18,19] obtained through the chitin esterification with butanoic anhydride proceeding in the presence of perchloric acid as a catalyst. DBC is soluble in typical organic solvents. DBC solubility in the organic solvents allowed the obtaining of fibre and nonwovens from DBC [19,20]. DBC biological properties tested in vitro and in vivo [21,22] show that, just like chitin, it is a biocompatible, bioactive polymer characterised by anti-allergic features, undergoing slow biodegradation and enhancing wound healing. Bifunctional chitin derivatives have been studied, expecting that introducing various substituents will improve/modulate the biopolymer performance features. Trichloroacetate-formate chitin ester was obtained in the reaction of chitin with formic acid and trichloroacetic acid [23,24]. It has biological properties similar to chitin, but their low solubility in organic solvents limited their practical application [25].

Van Luyen and Rossbach [26] proposed a method of obtaining both chitin mono-esters and chitin di-esters under the homogenic conditions by treatment of chitin solution in methanesulphonic acid with acetic acid anhydride and butyric acid anhydride, leading to the mixture chitin butanoate, chitin acetate, as well as mixed butanoate-acetate esters. It has been found that methanesulphonic acid can be substituted with trifluoroacetic acid [27]. The approach proposed by Bhatt et al. [28] in which the mixture of trifluoroacetic acid anhydride and orthophosphoric acid was applied to obtain in situ trifluoroacetic acid has been found to be the general method. This method was used for acylation of chitin with a variety of aliphatic acids: butanoic acid, cyclopropanecarboxylic acid, cyclobutanecarboxylic acid, cyclopentanecarboxylic acid, cyclohexanecarboxylic acid [29], and aromatic acids [30]. For butyryl derivatives, the high yield of esterification within the degree of substitution (DS) in the range 1.9–2.38 was observed, dependant on the excess of the use of butanoic acid.

Chitin modification based on the simultaneous incorporation of the acetyl and butyryl group to chitin leads to butyric-acetic chitin co-polyesters (BAC 90/10) soluble in typical organic solvents [31,32]. Biological tests of butyric-acetic chitin co-polyesters containing 90% of butyryl and 10% of acetyl groups confirmed the lack of allergic reaction and toxicity of material and also the ability to enhance the wound healing process without the need to change the dressing.

Promising preliminary results of the biological tests of butyric-acetic chitin co-polyesters 90/10 [33] encouraged us to develop a method of obtaining the porous structures of BAC 90/10, applying the method based on leaching the porophor agent. 

The main aim of our study was to develop a method of manufacturing porous materials useful for dressing of wounds which are hard to heal. The porous structure of the material arises from its manufacturing method consisting of pouring the solution of butyric-acetic chitin co-polyesters 90/10 on a layer of water soluble inorganic salts dispersed onto particles of defined diameter.

The research hypothesis assumes that after dissolving salt the obtained structures will resemble the shape of the porophor’s crystal, forming the open pores. The developed method of manufacturing the dressing materials will allow for their constant, repeatable, and automated production on an industrial scale.

Thus, the developed industrial method should enable the manufacture of the materials in the form of powder, membrane, and external membrane containing the bactericide. The membrane form should be adjusted to shallow wounds with a large surface area. Silver additive has bactericide activity for treatment of infected wounds, deep wounds, or slow-healing ones.

## 2. Materials and Methods

### 2.1. Materials

Butyric-acetic chitin co-polyester (BAC 90/10) used in the study is a trade product and is obtained on a production scale from Tricomed SA labs (Lodz, Poland) [34,35]. The molar weight of produced BAC 90/10 is determined on the basis of viscosimetric measurements, with establishing the intrinsic viscosity in dimethylacetamide (DMAc) at a temperature of 25 °C. BAC 90/10 used in the tests was characterised by viscosity within 1.8–2.4 dL/g. The inorganic salts used as the porophor agents are presented in Table 1. All salts were trade products with the appropriate analysis-purity (a.p.). The fractions of the molecule diameter within 0.16–0.4 mm were used.

Micro silver used in the process of obtaining the materials in the form of a membrane with bactericide activity was delivered by BioGate AG (Bremen, Germany). The material, according to manufacturer’s declaration, is compliant with the 76/768/EEC directive for cosmetics within the II, III (part 1 and 2), IV (part 1 and 2), VI (part 1), and VII (part 1) range. The organic solvents with analysis-pur (a.p.) were used as the trade products without any additional methods of purification.

### 2.2. Porous Material Preparation Using the Salt-Leaching Method—Laboratory Scale

In the trial tests it was found that the recommended method for forming thin porous dressings from butyric-acetic chitin co-polyesters (90/10) is to use the solution of 2–5 g of polymer in 100 mL of solvent. However, for a polymer with a viscosity of 2.2 dL/g, the optimum concentration was obtained by dissolving 3 g in 100 mL of solvent. Ethanol turned out to have the best features as a solvent, evaporating easily at 25 °C in the open air and, moreover, it is allowed to be used for medical purposes. The established density of butyric-acetic chitin co-polyesters solution was 0.9 g/mL.

The typical procedure of forming the dressings in the shape of porous films: chitin co-polyester containing the butyryl acid (90%) and acetic acid (10%) residues (BAC 90/10) was dissolved in ethyl alcohol, giving a 5% solution. Solid inorganic salt (6 g) was placed in a Petri dish with a 346 cm^2^ surface. The size of inorganic salt grains was within a 0.16 mm to 0.40 mm range (each salt was sieved before application). Five percent BAC 90/10 in ethanol was finally poured in 10 mL. After ethanol evaporation at a temperature of 40 °C in a sealed chamber (due to ethanol recovery) from the formed foil, inorganic salt was removed using deionised water. The washing process was run up to the moment when the inorganic salt disappeared in the water. After drying, in a temperature not exceeding 80 °C, the porous dressings with a thickness of 11 µm were obtained.

Instead of ethanol used to dissolve butyric-acetic chitin co-polyesters, other solvents with a higher density were tested. The mixture of ethanol (d = 0.789 g/mL) and chloroform (d = 1.48 g/mL) 35:65 (v:v) gave a transparent and homogenous solution with a density equal to 1.23 g/cm^3^. Application of a solvent with a density equal to 1.23 g/mL guaranteed a stable suspension of inorganic salt. An ethanol and chloroform mixture was used to get the suspension of the following salts: di-ammonium oxalate (6 g in 10 mL dilution), sodium carbonate (10-hydrate) (6 g in 10 mL dilution), and di-ammonium hydrogen citrate (6 g in 10 mL dilution).

A typical procedure for forming porous dressings using a slurry is described in the present paragraph. The salt suspension in the polymer solution, prepared according to the procedure previously described for suspension of the mixture, ethanol/chloroform with 60 g of the corresponding salt, and 100 mL of solvent using mechanical stirring for 10 min, was placed on a flat Teflon surface, then the slurry was spread over the surface using a blade, resulting in a thickness of 15 mm. The whole system was transferred to a closed dryer and the solvent was removed at 80 °C during a period of 4 h. After obtaining a dry film, the salt removal process was analogous to BAC 90/10 pouring the solution onto salt crystals.

### 2.3. Physico-Chemical Properties of Porous Materials Obtained on a Laboratory Scale

Scanning electron microscopy (SEM) was performed on porous films for the porosity characterisation. The specimens were sputter-coated with a palladium-gold alloy and analysed using a scanning electron microscope (Jeol, JSM-5400, JEOL Ltd., Akishima, Japan) operating at an accelerating voltage of 25 kV and desired magnification.

The pore size distributions were obtained by the SEM image analysis using ImageJ 1.52e (an open source image processing package based on ImageJ). Pore diameter in the images was compared with the magnification bar. For each polymer/salt mode two pictures were taken, measuring the diameter of 30 randomly chosen pores, according to the stereology methods, which is sufficient to confirm the size of the macro pores and their open character.

Porosity of the membranes was determined, using the equation [36]:

P = (1 − d*_s_*/d*_b_*) × 100

where: d*_s_*—density of porous dressing, d*_b_*—density of butyric-acetic chitin co-polyesters (1.35 g/cm^3^) determined by foil poured from the alcohol solution in compliance with the norm (ASTM D-792, Standard Test Methods for Density and Specific Gravity of Plastics by Displacement) using ethylene glycol as the solvent of known density.

Pore distribution was determined for selected variants by mercuric porosimetry with an Auto Pore IV 9500 apparatus (Micromeritics, Norcross, GA, USA). Prior to measurement, the samples were degassed to remove moisture, air, and solvent residue. Degassing was performed to obtain a vacuum of 15 μm Hg. The sample was subsequently flooded with mercury; because of the high contact angles and surface tensions, the mercury did not penetrate the porous structure of the sample. The incorporation of mercury into the sample pores was possible after increasing the pressure of the measurement system. Increasing the pressure enables pores with smaller diameters to be filled. The measurement consists of determining the relationship between the quantities of mercury incorporated (intrusion) and the pressure under which the intrusion occurs. The system allowed measurements at a maximal pressure of 413 MPa, which enabled determination of the porous structure of the porous dressing within the range of 400,000 to 3.5 nm. Based on the intrusion values obtained at successive pressure values, a curve of pore distribution was plotted for the sample in which the numerical values describe the porous structure: The total pore surface and average pore size.

### 2.4. Fabrication of Porous Chitin Butyric-Acetic Co-Polyester Useful as Wound Healing Material by Using NaCl Leaching Method—Enlarged Scale

**Figure 1:**
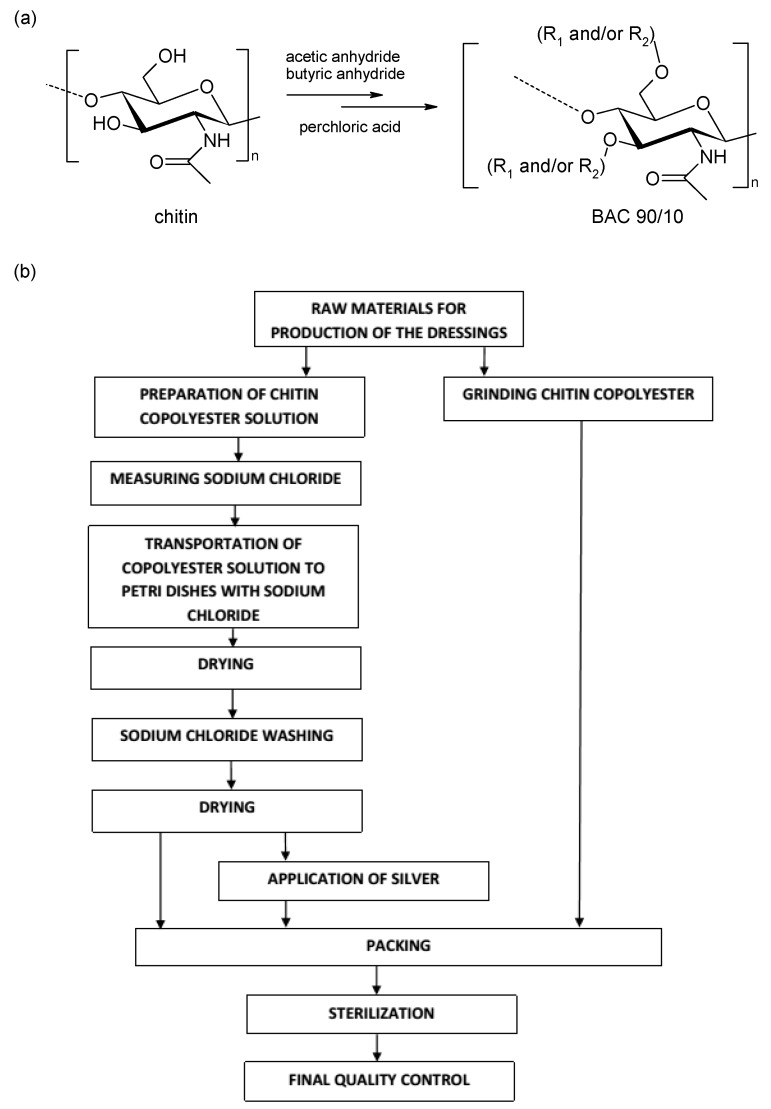
(**a**) Structure of chitin and BAC 90/10; (**b**) block diagram of manufacturing of the porous dressing materials, Medisorb series R Membrane, Medisorb R Ag, derived from BAC 90/10.

The typical process for obtaining porous dressing materials: 3% solution of butyric-acetic chitin co-polyesters 90/10 (BAC 90/10) in 96% ethanol was poured (7 mL on 1 dressing with diameter 9 cm) on the surface on which the layer of porophor agent is evenly distributed-sodium chloride (NaCl) (10 g on 1 dressing with diameter 9 cm). The materials used in production of porous dressing materials in the enlarged scale are presented in Table 2. After the solvent evaporation at a temperature of 40 °C, in the manner enabling the solvent recovery, the dressing material was transported to the box with the deionised water. Salt removal was continued (average five water changes were made) till the total chloride ions vanish in the washing media (the lack of chloride ions was confirmed by the absence of sediment precipitation against silver nitrate). Once the NaCl is removed, the resulting dressing materials were dried at a temperature of 80 °C (at reduced pressure for 4 h) in order to remove the water used in the process of porophor agent leaching. Porous dressing enrichment in the bactericide factor: The manufacturing process is analogical to the one described above with an additional phase of incorporation of the metallic silver on the surface in the form of micro molecules (micro silver) with bactericide characteristics confirmed. Silver is spread on the dressing material through spraying the silver suspension on the surface using a spray nozzle. Silver suspension is prepared by adding the metallic silver in powdered form of 1 g on 1 dm^3^ of water and then sonication of the blend using the ultrasound probe with 150 W power, frequency 40 kHz for 5–10 min, using the 2 cm diameter probe which is immersed in the solution 20 cm deep. Water excess is removed from the dressing in the drying process at 40 °C (at reduced pressure). The dressing materials, prepared in such a manner, are packed and submitted to the radiation sterilisation process in which the radiation dose is 25 kGy. Figure 1 presents the schematic method of producing the dressing materials from butyric-acetic chitin co-polyesters 90/10 with the use of NaCl as the porophor agent, using the salt-leaching method.

### 2.5. Physio-Chemical Parameter Tests for Porous Dressing Materials of Medisorb Series 

Water extracts taken from the manufactured porous dressing materials of the Medisorb series were compliant with the PN-EN ISO 10993-12 procedure. Extraction was run at a temperature of 37 °C and lasted 24 h. The water extracts underwent the following tests:

(1) Organoleptic analysis (internal procedure SOP-KJC.02) allows determination of the colour and clarity of the water solution.

(2) pH measurement (PN-EN ISO 3071:2007).

(3) Permanganate oxidisability (PN-P-04896:1984) relies on establishing the quantity of permanganate potassium corresponding to milligrams of oxygen consumed for oxidation of organic substances and some nonorganic compounds present in the water extract of porous dressing materials (boiling point, weakly acidic solution). 

(4) Spectroscopic characteristic in ultraviolet radiation (SOP-KJC.05) allows the performance of the test on the presence of selected function groups of organic substances in the water extract from tested porous dressing materials.

(5) Accurate conductivity (SOP-KJC.04) is measured with the Pure Water Test (PWT) instrumentation, HI 98308, manufacturer: Hanna Instruments (Olsztyn, Poland). Electrolyte accurate conductivity is the opposite to resistance expressed in micro-siemens per centimetre.

### 2.6. Biological Tests of Dressing Materials of Medisorb Series 

Dressings prepared from butyric-acetic chitin co-polyesters (90/10) (BAC 90/10) (Medisorb R Membrane) were used for biological tests. Square-shaped samples of 1.5 cm × 1.5 cm were used for studies. Cytotoxicity tests according the method described in PN-EN ISO 10993-5:2009 “Biological evaluation of medical devices. Cytotoxicity tests: in vitro methods” were done. All the tests were carried out with extracts (an indirect method).

*Cell line*: In the studies, referential cell line 3T3/Balb mice fibroblasts obtained from the Tissue Bank of Institute of Immunology and Experimental Therapy of the Polish Academy of Sciences in Wrocław, were used. The cells were stored in liquid nitrogen. After defrosting, the cells were passaged twice with a solution comprising 0.05% trypsin, 0.02% EDTA in phosphate-buffered saline (PBS) (pH = 7.2) (Sigma, St. Louis, MO, USA).

For cultivating the fibroblasts, a growth medium comprising Minimum Essential Media (MEM) Eagle (Sigma), 10% FCS (foetal calf serum, Sigma), glutamic acid (100 µg/mL, Sigma), penicillin (100 µg/mL, Sigma), and streptomycin (100 µg/mL, Sigma) was used. The cell culture was carried out in a Heraeus incubator (Thermo Scientific, Waltham, MA, USA), with the following parameters: 5% CO_2_, 37 °C, permanent humidification of the chamber.

*Preparation of extracts:* The tested Medisorb R material about the surface of 120 cm^2^ was treated with 20 mL of growth medium with serum and it was incubated at a temperature of 37 °C for 24 h. As a control group, a growth medium without the Medisorb R material was used, which was also incubated in the same conditions. 

*Performed tests:* The mice fibroblasts were placed in 12-well culture plates manufactured by NUNC (Nunc, Roskilde, Denmark), in each well, 0.5 × 10^6^ each. After 24 h, the cells adhered to the base and covered approximately 50% of the plate. The growth medium was then removed, and the tested Medisorb R material extracts or control extracts were added. The incubation was continued at 37 °C and in the 5% CO_2_ atmosphere. The cultures were assessed by reversed phase-contrast microscope after 24, 48, and 72 h. Medisorb R extracts were assessed on 9 cell cultures (3 for each day of the test).

*Cytotoxicity evaluation*: The quantitative and morphologic changes after contact with the tested Medisorb R material were assessed after 24, 48, and 72 h with the use of a reversed phase-contrast microscope. In order to determine the number of dead cells, trypan blue was used. The toxicity level was evaluated on the basis of the changes in morphology of the cells, their viability and ability to proliferate according to Table 3.

*Statistical analysis:* The results have been presented as a mean ± standard deviation (SD). The statistical analysis was carried out using one-way analysis of variance (ANOVA) using the Student *t* test. It was assumed that the correlation coefficient is significant when * *p* < 0.05, ** *p* < 0.01, *** *p* < 0.001.

## 3. Results and Discussion 

### 3.1. Manufacturing of Porous Dressing Materials from Butyric-Acetic Chitin Co-Polyesters 90/10 with Different Porophor Agent. Selection of the Most Useful Inorganic Salt Characterised by Porophor Agent Activity

At the stage of trial tests, it was stated that the typical procedure of manufacturing porous dressing materials from butyric-acetic chitin co-polyesters 90/10 has to guarantee obtaining the films with open pores whose diameter should not exceed 0.4 mm. The process of finding the most accurate way of forming porous membrane involved finding the method of pouring the membrane from suspensions. Taking into consideration the low density of butyric-acetic chitin co-polyesters 90/10 3–5% in ethanol, preparation of stable suspensions of inorganic salts in a solution of BAC 90/10 was impossible, because the inorganic salts used as a porophor agent were quickly falling to the bottom of the dish. There were attempts to change the density by preparing the solution of butyric-acetic chitin co-polyesters 90/10 in the mixture 35:65 (v:v) of ethanol (d = 0.789 g/mL) and chloroform (d = 1.48 g/mL). Finally, a transparent and homogenous solution with a density of 1.23 g/mL was obtained. The use of butyric-acetic chitin co-polyesters 90/10 in the mixture ethanol-chloroform enabled a stable suspension from: di-ammonium oxalate monohydrate (6 g in 10 mL solution) giving a solution with a density of 1.48 g/mL, sodium carbonate (10-hydrate) (6 g in 10 mL solution) with a density of 1.46 g/mL, di-ammonium hydrogen citrate (6 g in 10 mL solution) with a final density of 1.48 g/mL. From the obtained stable suspensions, the membranes were formed, and after the drying process and the removing of inorganic salt, they were characterised by the satisfactory porosity and pore size (diameter below 0.4 mm). Taking into account that in the procedure chloroform is used, that method does not seem to be proper for dressing production on an industrial scale. It was stated that obtaining the stable suspension in the polymer solution in ethanol would be possible using NaHCO_3_ as a porophor agent. The stable in time suspension was obtained from a 3% solution of butyric-acetic chitin co-polyesters 90/10 in ethanol (8 mL and 4.5 g (53.6 mmol) sodium bicarbonate). The suspension was stable for 2 h. In the experiment, in which the porophor agents NaHCO_3_ or KHCO_3_ were used and citric acid (2 mmol of citric acid per 3 mmol of hydrocarbonate), CO_2_ evolution was observed, which significantly shortened the time of inorganic salt removal from the material. The CO_2_ rapid secretion led to, on one hand, membrane separation from the surface on which it was formed, which made the cleaning process more effective, but on the other hand, it reduced mechanical properties of the obtained structures through micro-damage of the inner walls which are the constructive element of the porous membrane.

Searching for the optimum ratio of added porophor agents, which ensure the high porosity of the dressing, led to the conclusion that the optimum is to use 30–60 g of inorganic salt with the grain size of 0.16–0.40 mm (each salt was sieved before application) on 100 mL of polymer solution. In standard conditions, 10 mL of polymer solution was poured on the 10 cm^2^ Petri dishes, on which 3–6 g of inorganic salt was placed. After the alcohol evaporated, the foils were formed, from which the inorganic salt was removed using deionised water. It enabled the porous dressing materials to get a thickness between 8 and 11 mm.

### 3.2. Porosity of Materials Obtained from Butyric-Acetic Chitin Co-Polyesters 90/10

The use of inorganic salt soluble in water with a different bulk density resulted in formation of an open pore structure with many thin-wall polymer connectors. The porosity study, as shown in Table 4, indicated that the obtained materials are highly porous with a porosity level up to 99%. Such high porosity of foils were characterized as moderately high in mechanical strength. 

The average pore size was determined by mercury porosimetry which shows the pore diameter measured in the hole through which mercury is introduced into the interior of the pores. In the case of the occurrence of the so-called open-bottle pore where the inlet is smaller than the maximum pore diameter, mercury porosimetry takes into account in the measurement of the average pore diameter, the pore diameter through which the measurement is made. Therefore, the determined value of the average pore diameter obtained with the mercury porosimetry has a different value than that obtained with the stereology method performed on SEM images.

The mechanical tests showed that for all manufactured high porous films the value of tensile strength for samples 2 cm wide and 0.11 mm thick was not more than 5 cN. There is a possibility to obtain better mechanical properties of the formed foils by reducing the porosity volume, which can be done by reducing the amount of porophor agent in ratio to the polymer and the use of a higher polymer concentration. The use of 3% solution of butyric-acetic chitin co-polyesters 90/10 in ethanol results in forming very thin walls connecting the pore structure, which has a negative impact on the mechanical properties.

**Figure 2:**
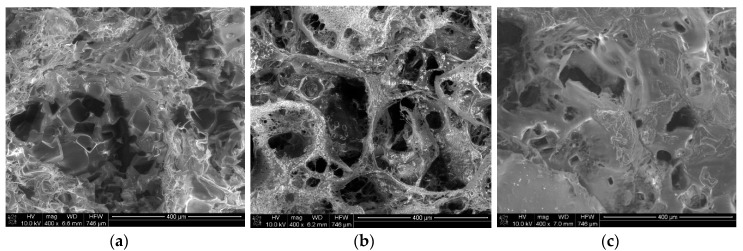
The SEM picture of the porous materials formed via the salt-leaching method from 3% (**a**), 4% (**b**), and 5% (**c**) solution of butyric-acetic chitin co-polyesters 90/10 in ethanol. The mass ratio of inorganic salt and the solution of BAC 90/10 was 6/10.

Pictures of the porous materials formed via the salt-leaching method from a solution of butyric-acetic chitin co-polyesters 90/10 are presented in Figure 2. SEM tests prove the presence of the pores in the shape of inorganic salt crystals. Microscope tests confirmed the presence of the open pore structure.

**Figure 3:**
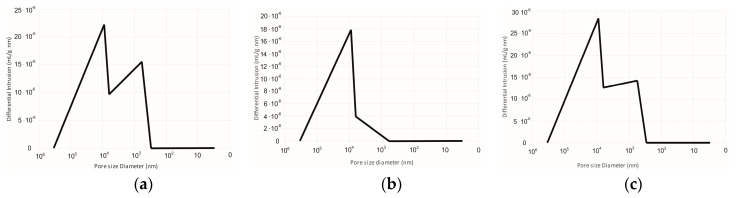
Chart presenting the layout distribution of the pore size of the material obtained by the salt-leaching method from 3% (**a**), 4% (**b**), and 5% (**c**) of butyric-acetic chitin co-polyesters 90/10. The mass ratio of inorganic salt to solution of BAC 90/10 in ethanol was 6/10.

Figure 3 shows a chart presenting the pore layout in the sample manufactured with the salt-leaching method using NaCl as a porophor agent at various polymer concentrations. The salt particle size was 0.16–0.40 mm.

The porosity tests presenting distributions of pore size of films made of butyric-acetic chitin co-polyesters 90/10 poured on salt crystals with diameter of 0.16–0.4 mm show two groups within pore size in the final material, as shown in Figure 3. The first one relates to porophor agent diameter and those pores can be described as big. The presence of pores of such type allows good liquid permeability through the manufactured material. The second group of observed pores is significantly smaller (pore diameter below 1000 nm, just like a micropore). Simultaneous presence of big, micro, and mezzo pores can stem from the method of material production. Big pores are modelled on the size of the used porophor agent in the shape of inorganic salt crystals, whereas the pores with diameters below 1000 nm are made as a result of the simultaneous process of polymer and solvent diffusion of solvent (ethanol) during the drying time of poured films. Total established pores density for the tested film samples was between 6.734 and 7.174 mL/g, while the external pore surface for produced films is within 0.578–0.612 m^2^/g.

### 3.3. Crystallinity Degree of Porous Materials 

High porous materials manufactured by the pouring method were not submitted to any ridding processes, e.g., stretching, like in the classic techniques in foil or fibre forming. That is why in the manufactured structures there is no macromolecule orientation. Crystallinity degree is set up for formed high porous materials and was equal to 27.3% for film poured from the ethanol solution of butyric-acetic chitin co-polyesters 90/10 and 27.2% for films poured from the mix of solvents ethanol/chloroform, independently of the porophor agent use, as shown in Table 5. The value originates from the fact that the polymer has the ability to organise itself in space while forming the gel form during the solvent removal process.

The determined crystallinity degree for porous films are pressingly lower than in the case of fibres formed from chemically similar butyric-acetic chitin co-polyesters 90/10 where the butyryl group is 95% and the acetyl group is 5%. It can stem from the way of forming these type of structures.

### 3.4. Porous Materials in the Form of Film Obtained in the Enlarged Scale. Medisorb Series Products

Optimal experiment conditions on a laboratory scale enabled R&D research to adapt to a bigger scale. A typical manufacturing process runs in such a way that butyric-acetic chitin co-polyesters 90/10 is poured on the porophor agent surface, then the solvent evaporates and the porophor agent is removed from the dressing structure. In this process, 3% of butyric-acetic chitin co-polyester 90/10 (BAC 90/10) is used in 96% ethanol. The solvent is poured on the evenly spread porophor agent layer (sodium chloride). The solvent evaporates at a temperature of 40 °C, so that it enables its condensation. Once the ethanol fully evaporates, the material is treated with deionised water. The salt-removing process is run till the moment when all chloride ions disappear. The last stage is drying at a temperature of 80 °C in order to remove the water used in the stage of salt leaching. Dressing materials obtained with the salt-leaching method in the form of porous films are in the shape of the petal which is characterised by a high porous structure, similar to a sponge. The porous material is enriched with a bactericide factor which is manufactured analogically to the process mentioned above. There is an extra stage of applying micro silver in the form of micro-molecules (micro silver) on the surface. Silver proves to have an antibacterial effect. Silver is put on the dressing through spraying the silver suspension on the dressing surface using a spraying nozzle. Silver suspension is prepared by adding the metallic silver in the amount of 1 g per 1 L water, then the process of substance sonification occurs with the use of the ultrasound device. The water excess is removed from the dressing in the drying process at a temperature of 40 °C.

The manufactured materials were tested in terms of chemical parameters. The procedures and ISO (International Organization for Standardization) norms were used, alongside with the internal procedures. Water extraction from the porous materials of the Medisorb series was compliant with PN-EN ISO 10993-12. The extraction was done at a temperature of 37 °C and lasted 24 h. The achieved results for chemical parameters are presented in Table 6.

**Figure 4:**
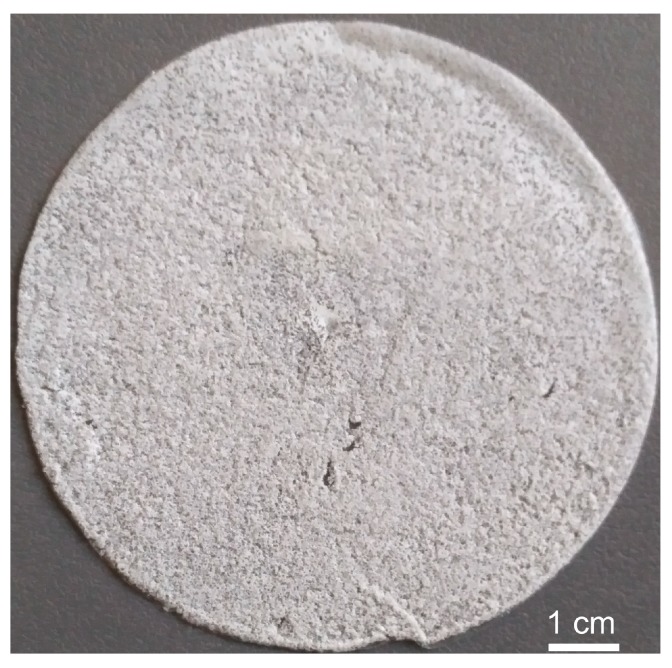
The porous material from butyric-acetic chitin co-polyesters 90/10 and NaCl as the porophor agent in the membrane form.

**Figure 5:**
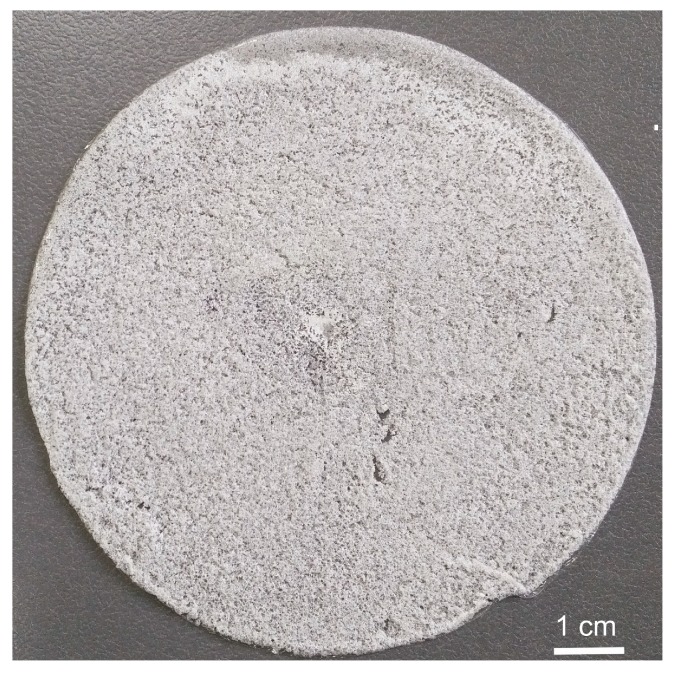
The porous dressing material from co-polyester BAC 90/10 and NaCl as the porophor agent in the form of porous membrane with the bactericide substance (micro silver particle).

Water extracts obtained from dressing materials must be transparent, colourless, with pH in the range from 4 up to 7. The results presented in Table 5 point out that the tested materials meet the requirements. Permanganate oxidisability results are much lower than the set limit (maximum 2.5 mg O_2_/g). Maximum absorbance in the ultraviolet radiation meets the requirements for implanted materials (maximum 0.3 nm). Accurate conductivity shows a low rate. The achieved results confirm the chemical purity of the dressing materials of Medisorb R and Medisorb R Ag. The porous materials are manufactured in two forms: as the porous membrane, as shown in Figure 4, and as the porous membrane which contains the bactericide substance (micro silver), as shown in Figure 5.

**Figure 6:**
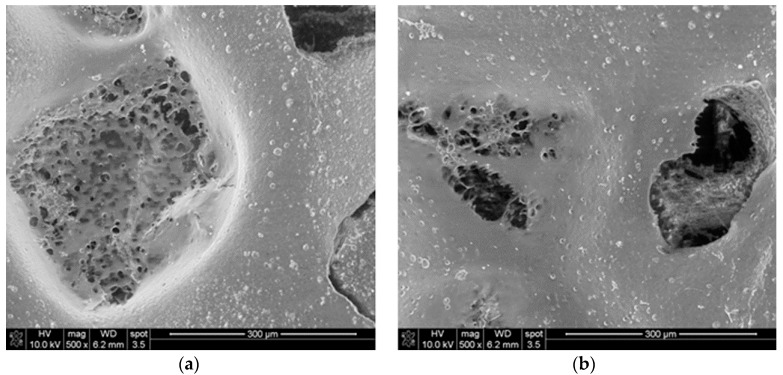
The SEM picture of the porous structure obtained as a result of the salt-leaching method in the enlarged scale. Polymer concentration 5%, polymer solution to salt mass ratio 10/6, solvent-ethanol, salt-natrium chloride with particle diameter 0.16–0.40 mm. (**a**) Imaging of the shape of porophor’s crystal; (**b)** documents the open pore character.

Dressing materials presented in Figure 4 and Figure 5 were manufactured according to the salt-leaching method with the use of butyric-acetic chitin co-polyesters 90/10 with the viscous volume 1.4 dL/g, sodium chloride with a molecule diameter of 0.16–0.40 nm, and micro silver particles. Figure 6 presents the SEM picture of the porous membrane structure produced with the method of pouring the polymer solution on the salt crystals. The picture analysis states that the pore shape is a negative-copy of the inorganic salt crystal. Moreover, it can be stated that the obtained pores are open pores, which influences the adsorption of wound effusion.

Porous materials of butyric-acetic chitin co-polyesters 90/10 manufactured with the method of pouring the dilution of polymer on solid NaCl, with a defined molecule size obtained in the enlarged scale, are characterised by high repetitiveness of all crucial parameters which confirms the repetitiveness of the manufacturing method. The selected volumes for middle-size pores for the production series are within 290 µm ± 15 µm, as shown in Table 7.

The salt-leaching method for forming high porous dressings from butyric-acetic chitin co-polyesters 90/10 leads to obtaining the porous materials whose pores shape is modelled on the used salt crystals. The character of the forming process in the salt-leaching method is characterised by loose solvent evaporation, which results in obtaining a lower crystallinity degree of the forms in comparison to the process of fibre formation. For high porous films, the average crystallinity volume was reached, and it was equal to 27.3%. The difference in the crystallinity volume probably stems from the lack of the salt-leaching process, and a physical influence on the polymer during its solidification.

It was confirmed that material Medisorb R does not show cytotoxic activity. The tests were performed according to direct contact methodology, which is compliant with the PN-EN ISO 10993-5:2009 norm, issued by the National Medicine Institute, which is the authorised agency testing medical devices.

### 3.5. Assessment of Cytotoxic Activity of Medisorb R Material

The proliferation studies on 3T3/Balb mice fibroblasts in the presence of Medisorb R extract lead to the conclusion that the tested material is not cytotoxic, as shown in Table 8.

**Figure 7:**
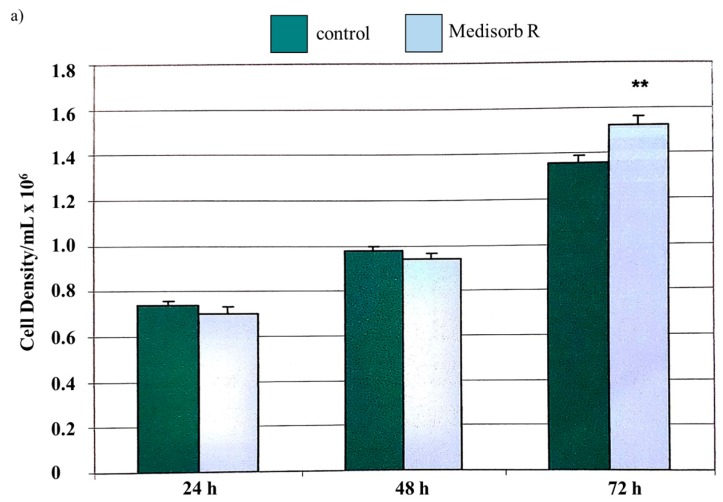

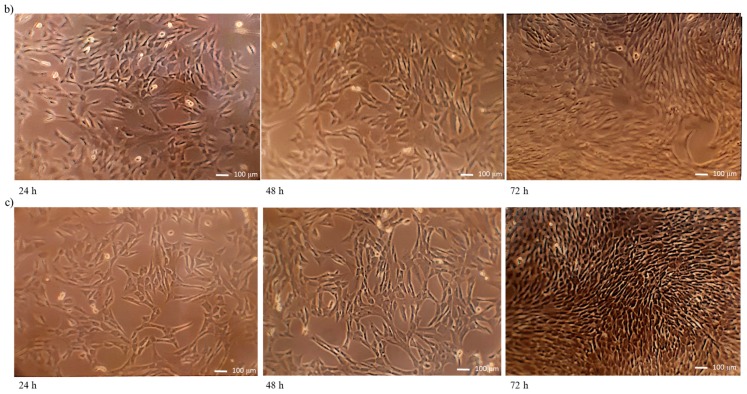
(**a**) Proliferation of 3T3Balb/C mice fibroblasts after 24, 48, and 72 h the presence of Medisorb R extract. Microscopic pictures of T3TBalb/C mice fibroblasts cells: (**b**) control culture (**c**) culture with Medisorb R extract. Magnification 100×.

There was no difference between the cell proliferation capacity in the presence of the tested material and the control sample. It was found that Medisorb R has a positive influence on cell proliferation, as shown in Figure 7a, after 72 h.

Based on the microscopic pictures evaluation, it was stated that in the case of the control culture, cultures after 24, 48, and 72 h, the cells adhered and had normal morphological features, as shown in Figure 7b. No agglutination, vacuolation, or unsticking from the medium or lysis of cell membranes were observed. The cells proliferation was at a normal level. After 72 h, the dead cells amount was under 1%. 

Figure 7c shows the cells had normal morphological features in all tests performed on Medisorb R extract. Similar to data obtained for extract control culture, no agglutination, vacuolation, or unsticking from the medium or lysis of cell membranes were observed. After 24 and 48 h, there was an insignificantly lower cell proliferation level; however, after 72 h, the proliferation was significantly higher when compared with control cultures. After 24 and 48 h, no dead cells in the culture were observed. The percentage of dead cells was identical in the extracts, as well as in the control group, after 72 h.

## 4. Conclusions

A method of obtaining high porous materials in the form of film using butyric-acetic chitin co-polyesters 90/10 with butyryl group 90% and acetyl group 10% was developed. High porous films of 0.11 mm thickness were obtained with two methods. The first method was based on pouring the 5% BAC 90/10 solution in ethanol on the flat, glass surface which was covered with inorganic salt. The ratio mass of the salt dilution was 10/6. In the second method, the salt suspension (6 g in 10 mL) in the BAC 90/10 solution was poured on the glass surface. Obtaining the time-stable inorganic salt suspension in the organic solution of BAC 90/10 requires the composition of the solvent mixture density similar to inorganic salt bulk density. The optimum solution was achieved by mixing ethanol with chloroform. Both methods for obtaining the porous films of butyric-acetic chitin co-polyesters 90/10 allowed the gaining of high porous materials, with the porosity volume of 95–99%. With such high porosity, the films were characterised by mechanical properties of tensile strength to the level of 5 cN. Films with such durability parameters and porosity level can be accepted to use as an active layer of medical dressings. The method for forming the porous film includes pouring polymer solution on the layer of sodium chloride used as a porophor agent and evaporation of the organic solvent, which results in obtaining the materials with a lowered crystallinity level compared to others formed from butyric-acetic chitin co-polyesters 90/10 with 95% butyryl groups and 5% acetyl groups. In the case of foil, the estimated crystallinity degree is 27.3%, while for fibre it is 35% because of the additional stages in enhancing the orientation of the polymer matrix that occurs there. The optimised method for obtaining the porous dressing materials (Medisorb series) includes the use of NaCl as the porophor agent in the form of the solid layer and 3% solution of butyric-acetic chitin co-polyesters 90/10 in ethanol. It enables the obtaining of the materials which are characterised by high porosity and chemical purity confirmed by organoleptic analysis pH tests, permanganate oxidisability, absorbance in the ultraviolet radiation, and the accurate conductivity of water extracts. The results of the permanganate oxidisability and the absorbance in the ultraviolet radiation prove a very low amount of contamination, below the level acceptable for medical materials. Moreover, Medisorb R materials show no cytotoxic activity in vitro.

## Figures and Tables

**Table 1:** The applied inorganic salts. The data in the table are taken from the safety data sheets provided by the manufacturer of each reagent.

Name of Porophor Agent	Chemical Formula	Bulk Density [g/mL]	Solubility in Water at 20 °C [g/100g]
Potassium carbonate (Aldrich)	K_2_CO_3_	2.43	111.7
Potassium bicarbonate (Aldrich)	KHCO_3_	2.17	22
Potassium bisulphate(Aldrich)	KHSO_4_	2.20	51.4
Potassium nitrite (Aldrich)	KNO_2_	1.92	298
Ammonium carbonate (Aldrich)	(NH_4_)_2_CO_3_	1.50	32
Ammonium bicarbonate (Aldrich)	(NH_4_)HCO_3_	1.58	22
Ammonium bicarbonate 1-hydrate (Aldrich)	(NH_4_)HCO_3_·H_2_O	-	120 (15 °C)
Diammonium hydrogen phosphate (Aldrich)	(NH_4_)_2_HPO_4_	1.62	-
Ammonium sulphate (Aldrich)	(NH_4_)_2_SO_4_	1.77	75.4
Di-ammonium hydrogen citrate (Aldrich)	C_6_H_14_N_2_O_7_	1.48	100
Di-ammonium oxalate monohydrate (Aldrich)	C₂H₈N₂O₄·H₂O	1.48	45
Sodium carbonate 10-hydrate (Aldrich)	Na_2_CO_3_·10H_2_O	1.46	21.5; 421 (100 °C)
Sodium carbonate (Aldrich)	Na_2_CO_3_	2.53	21.5; 45.5 (100 °C)
Sodium bicarbonate (Aldrich)	NaHCO_3_	2.20	9.6
Di-hydrogen phosphate (Aldrich)	Na_2_HPO_4_	1.92	112
Sodium thiosulphate pentahydrate (Aldrich)	Na_2_S_2_O_3_·5H_2_O	1.73	32.8
Sodium chloride (Avantor Performance Materials)	NaCl	2.16	36

Aldrich: St. Louis, MO, USA; Avantor Performance Materials: Radnor, PA, USA.

**Table 2:** Characteristics of the material used in production of porous dressing materials in the enlarged scale. Medisorb series.

Material	Characteristics
butyric-acetic chitin co-polyester (BAC 90/10)	Material manufactured by Tricomed SA in compliance with the patent number P-400256 and 220238. A polymer having a viscosity of 2.2 dL/g was obtained according to the patent procedure.
Sodium chloride, analysis-purity (a.p.), grain size fraction 0.16–0.4 mm	Used as a porophor agent in the production of dressing materials in the form of membrane (Medisorb R Ag, Medisorb R Membrane); manufacturer: Avantor Performance Materials.
Micro silver	Used in the production of dressing material Medisorb R Ag, Material in accordance with the manufacturer declaration BioGate AG- MicroSilver BG Pharma (Bremen, Germany) —In compliance with the 76/768/EEC directive concerning cosmetics within II, III range (part 1 and 2), IV (part 1 and 2), VI (part 1), VII (part 1) —None of the substances that are elements of the composition show a cancerogenic, mutagenic, or toxigenic reaction. Micro silver is widely used in the production of cosmetics (cream, shampoo, toothpaste, etc.) and the production of medical devices (dressings, orthopaedic prosthesis, stents, etc.).
Ethanol 96% material purity	Commercial product, manufacturer: Kompania Spirytusowa Nord-Clas Sp. z o.o. (Lodz, Poland)

**Table 3:** Toxicity degrees in direct contact tests.

Degree	Toxicity	Changes in the Culture
0	none	single, intracytoplasmatic granularities, no lysis of the cells
1	light	20% of the cells are rounded, shrunk, unsticking from the growth medium without cytoplasm densities, single cells with damaged cell membrane
2	moderate	50% of the cells are rounded, without granularities, vast lysis of the cells, empty space between cells
3	medium	70% of the cells are rounded, lysis of the cells
4	strong	the cell culture is almost completely destroyed

**Table 4:** The results for middle-sized pores and total porosity for manufactured membranes with the use of various inorganic salts.

Porophor Agents	Organic Solvent	Stereology Pores Diameter [µm]	Hg Porosimetry Average Pores Diameter [μm]	Total Porosity [%]
Ammonium carbonate	ethanol	250	80	96–98
Sodium carbonate	ethanol/chloroform	250	120	96–98
Sodium bicarbonate	ethanol	250	70	96–99
Sodium dihydrogen phosphate	ethanol	260	100	96–98
Potassium bicarbonate	ethanol	260	70	96–98
Potassium carbonate	ethanol	270	110	96–98
Potassium nitrite	ethanol	270	90	96–98
Ammonium bicarbonate	ethanol	280	100	97–99
Sodium carbonate	ethanol	280	120	96–98
Ammonium bicarbonate 1-hydrate	ethanol	290	125	96–98
Sodium chloride	ethanol	290	95	95–98
Potassium bisulfate	ethanol	300	100	95–98
Ammonium sulfate	ethanol	310	90	96–98
Di-ammonium hydrogen citrate	ethanol/chloroform	320	115	96–99
Sodium thiosulfate	ethanol	320	110	96–99
Diammonium hydrogen phosphate	ethanol	340	130	95–97
Di-ammonium oxalate 1-hydrate	ethanol/chloroform	340	120	95–98

**Table 5:** The scale of the crystallinity of films from butyric-acetic chitin co-polyesters 90/10 manufactured using the method of pouring on the layer of inorganic salt crystals and poured in the form of suspension of salt in the polymer solution.

Sample	Crystallinity Value [%]
Porous film poured from ethanol	27.3
Porous film poured from mixture of ethanol/chloroform	27.2
Fibres of co-polyester (BAC 95/05)	35.0

**Table 6:** Chemical test results of porous dressing materials, Medisorb series.

Parameter	Methodology	Results	
		Medisorb R	Medisorb R Ag
Organoleptic analysis of water extract	SOP-KJC.02	Transparent colourless	Transparent colourless
pH tested sample [pH unit]	PN-EN ISO 3071:2007	5.63	6.04
Permanganate oxidisability [mg O_2_/g]	PN-P-04896:1984	0.540	1.022
Maximum absorbance in ultraviolet radiation within 230 nm [λ (nm)]	SOP-KJC.05	0.0729	0.0921
Characteristic volume within 245 nm [λ (nm)]		0.0685	0.0848
Accurate conductivity [µS/cm]	SOP-KJC.04	11.9	20.8

**Table 7:** The results of middle-sized pore measurements for the series of materials manufactured on an industrial scale.

Number of Sample Medisorb R Membrane	Middle-Sized Pores [mm]	Crystallinity Rate [%]
1	285	27.3
2	280	27.2
3	275	27.4
4	287	27.3
5	300	27.3
6	305	27.4
7	295	27.2

**Table 8:** Cytotoxicity test results of Medisorb R material in terms of 3T3/Balb mice fibroblasts.

Culture	Cell Density/mL × 10^6^	% of Dead Cells	Toxicity Degree
Test after 24 h			
Control culture	0.74 ± 0.02	0	0
Culture with Medisorb R extract	0.7 ± 0.02 *p* = 0.09	0	0
Test after 48 h			
Control culture	0.98 ± 0.023	0	0
Culture with Medisorb R extract	0.94 ± 0.0.28 *p* = 0.19	0	0
Test after 72 h			
Control culture	1.36 ± 0.03	1	0
Culture with Medisorb R extract	1.52 ± 0.35 ** p = 0.0039	1	0

* *p* < 0.05, ** *p* < 0.01, *** *p* < 0.001.
